# The Impact of the COVID-19 Outbreak on Lifestyle-Related Behavior Among the General Population

**DOI:** 10.7759/cureus.45756

**Published:** 2023-09-22

**Authors:** Vandna Pandey, Remiya Mohan, Ashok Kumar, P Gangadevi, Nancy Kurien

**Affiliations:** 1 College of Nursing, All India Institute of Medical Sciences, Jodhpur, IND

**Keywords:** covid-19, eating, sleep behavior, physical activity, lifestyle

## Abstract

Introduction: Coronavirus has affected more than 200 countries around the world. Due to lockdowns, people have limited outdoor activities and started adopting new and healthy lifestyle behaviors.

Method: A cross-sectional study was conducted to explore the impact of COVID-19 on lifestyle-related behavior. Data were collected from different regions of North India using Google Forms (Google, Inc., Mountain View, CA, USA) with the help of a standardized questionnaire. Both descriptive and inferential statistics were used for analysis. The p-value was set at <0.05.

Results: A total of 468 responses were recorded (mean age: 28.51 years). A significant improvement was found in the intake of fruits and vegetables as well as pulses, eggs, or meat during the COVID-19 pandemic. Participation in household chores and screen time in watching mobile or television were significantly increased. The majority (44.4%) of participants had stable weight, and 37.8% gained weight during the COVID-19 pandemic. The anxiety they felt in a day also increased, which was predominantly due to fear of COVID-19. During the COVID-19 pandemic, a significant association was found between physical activity scores and gender, residence, nature of work, and hours of work of study subjects.

Conclusion: There is an improvement in healthy eating behavior, quality of sleep, and participation in household chores during the COVID-19 pandemic. Further, domains of lifestyle can be explored to bring a positive style of living for the achievement of healthy lifestyle behavior by the general population.

## Introduction

The novel coronavirus outbreak began in December 2019. Coronavirus spreads rapidly from person to person. The World Health Organization declared COVID-19 as a pandemic on March 11, 2020 [1]. After that, the coronavirus pandemic continued to transform and affect the daily lives of communities worldwide. It has affected more than 200 countries around the world. Many governments adopted emergency measures such as total lockdowns and travel restrictions to prevent further transmission of infection. These restrictions help minimize the rate of infections. Such limitations negatively affected individuals by limiting participation in normal daily activities, sticking to a physical fitness regime due to closed gym, having no group gatherings, and increasing social distancing. Due to these lockdowns in several countries, people face limited access to participate in outdoor activities or avoid outdoor activities entirely [2].

Lockdown and travel restrictions also impose a burden on an individual’s health by compromising physical fitness, which strongly affects the ability to cope with infections and compromises the immunologic system. The most important risk factors for morbidity from major illnesses are physical inactivity and poor mental health. This is applicable to the general population and specifically to chronically ill populations, as well as older adults at the highest risk of COVID-19-induced mortality [3].

It is noteworthy that healthy lifestyles enhance the immune system, reduce the risk of respiratory infections and inflammation, and are effective in the prevention of many chronic conditions that increase the risk of severe COVID-19 infections. In addition, physical activities are beneficial in preventing anxiety and depression in stressful situations. The COVID-19 pandemic not only adversely affected the physical health of individuals but also brought significant positive changes in their lifestyle behavior [4].

The COVID-19 outbreak has impacted the daily life of many people. To limit the spread of COVID-19 and prevent disease severity, people started changing their lifestyles, especially their eating habits, and getting involved in physical activities. The intervention to stay at home, limitations of social interactions, and self-isolation changed the daily routine of individuals [5].

The lockdown intervention succeeded in reducing the exponential transmission of COVID-19 and resulted in positive health outcomes. However, major lockdowns have also led to other changes in life. Many studies have been conducted to highlight the negative impact of COVID-19 on physical and mental health, but recent studies found some positive lifestyle changes among adults, such as improvement in healthy eating behavior, more physical activities, and increased quality time with family [6].

Since the cure for COVID-19 is not yet known, optimizing the host immune system should be a vital strategy to combat the disease, limit its complications, and reduce mortality. The practice of a healthy lifestyle, such as a healthy diet, regular physical activity, adequate restorative sleep, good stress management, avoidance of tobacco and harmful substances, positive psychological well-being, and healthy social connections with friends, family, and colleagues, can significantly improve the efficacy of the immune response to diverse diseases, especially those of viral origin such as COVID-19 [7]. While countries are grappling with the imminent dangers that this virus poses to humanity, people started following a few key measures that can fight this pandemic [8].

Washing hands frequently, using an alcohol sanitizer to disinfect hands, wearing a mask to cover the nose and mouth, and avoiding touching the mouth or nose with dirty hands have become hygiene standards for people. Certain methods to improve immunity are also paramount at this juncture [9].

There is a strong positive correlation between a healthy lifestyle and enhanced immune function. Also, there are relationships between lifestyle modification and immune system improvement. Available evidence suggests that healthy lifestyle practices among patients with infectious diseases, especially those of viral origin, may boost their immune systems and shorten the duration of their disease. Evidence also exists suggesting that some components of an unhealthy lifestyle (such as poor diet, physical inactivity, stress, smoking, alcohol, loneliness, and poor sleep) may significantly impair the immune system and predispose people to greater susceptibility to infectious diseases [10].

Thus, we felt the need to conduct a cross-sectional study to evaluate the overall impact of the COVID-19 outbreak on lifestyle-related behavior.

## Materials and methods

A quantitative research approach and a cross-sectional study design were adopted in the present study. The research variable of the study was lifestyle-related behavior. The demographic variables of the study were age, educational status, occupation, type of family, family income, residential area, job profile, and nature of work. The inclusion criteria for the sample were those in the age group above 18 years, able to speak and understand Hindi or the English language, and willing to participate in the study; those who did not meet these criteria were excluded. The sample size calculated for the study was 450 using the following formula:

n={DEFF*Np(1-p)}/ {d2/z2 1-α/2*(N-1) +p*(1-p)}.

The sample taken for the study was 468.

Data collection tool

The tools for data collection were a demographic data sheet and a standardized questionnaire by Chopra et al. [11] to evaluate the impact of COVID-19 on lifestyle-related behavior. The internal consistency of the tool was 0.83. The questions were prepared in Hindi and English language. Written permission to use and create the tool using Google Forms (Google, Inc., Mountain View, CA, USA) was obtained from the author. Data were collected by interview technique or online survey as feasible from July 2021 to December 2021. The Google Forms link was created and circulated via emails and WhatsApp (Meta, Menlo Park, CA, USA).

Ethical consideration

Before commencing the study, ethical clearance was obtained from the Ethics Committee of All India Institute of Medical Sciences, Jodhpur (AIIMS/IEC/2021/3543). Informed consent was obtained from all respondents. The information about the person to be contacted for queries was provided on the first page of the questionnaire and Google Forms.

Statistical analysis

The collected data was analyzed using Statistical Package for the Social Sciences (SPSS) version 20 (IBM SPSS Statistics, Armonk, NY, USA). Descriptive and inferential statistical measures such as frequency, percentage, mean, standard deviation (SD), mean difference, paired t-test, independent t-test, analysis of variance (ANOVA), and post hoc test were used to analyze the data. For all analyses, a p-value of <0.05 was considered statistically significant.

## Results

Demographic characteristics

A total of 468 responses were collected using Google Forms. The mean age of the participants was 28.51 years. Among the participants, 42.3% were male and 57.7% were female, 56% were unmarried, and 60% were residing in a nuclear family. The majority of the participants (82.5%) graduated from a university. Only 7.3% have a family income of less than 10,000 Rupees, and 55.1% are residing in urban areas. Nearly 30.8% were performing long-standing work, 61.5% were doing 6-8 hours of work per day, and only 7.9% were suffering from any illness. Many of the participants (44.4%) maintained a stable weight, whereas 37.8% gained some weight during the COVID-19 pandemic (Table 1).

**Table 1:** Demographic characteristics of the study participants (N=468) COVID-19: coronavirus disease 2019, SD: standard deviation

Sample characteristics	Frequency (%)
Age (years) (mean±SD)	28.51±8.31
Up to 30 years	309 (66)
31-60 years	159 (34)
Gender	
Male	198 (42.3)
Female	270 (57.7)
Educational status	
No formal education	1 (0.2)
Secondary	5 (1.1)
Higher secondary	76 (16.2)
Graduate and above	386 (82.5)
Marital status	
Married	201 (42.9)
Divorced	5 (1.1)
Unmarried	262 (56)
Type of family	
Nuclear	281 (60)
Joint	183 (39.1)
Extended	4 (0.9)
Family income (Rupees)	
Below 10,000	34 (7.3)
10,000-30,000	84 (17.9)
30,000-50,000	87 (18.6)
50,000-70,000	97 (20.7)
More than 70,000	166 (35.5)
Residential area	
Rural	113 (24.2)
Semi-urban	97 (20.7)
Urban	258 (55.1)
Nature of work	
Office work	127 (27.1)
Field work	44 (9.4)
Long-standing work	144 (30.8)
Computer/sedentary work	68 (14.5)
Household work only	28 (6)
Others	57 (12.2)
Working hours per day	
Below 6 hours	64 (13.7)
6-8 hours	288 (61.5)
More than 8 hours	116 (24.8)
History of chronic disease	
Yes	37 (7.9)
No	431 (92.1)
Weight gain during COVID-19	
Stable	208 (44.4)
Lost	71 (15.2)
Gained	177 (37.8)
I don’t know	12 (2.6)

Comparison of lifestyle behavior before and during COVID-19

Lifestyle behavior was assessed on various parameters such as eating behavior, physical activity, sleep patterns, and other lifestyle behaviors such as smoking habits, alcohol consumption, and family and friends’ support for maintaining a healthy lifestyle (Table 2).

**Table 2:** Comparison of lifestyle behavior before and during COVID-19 (N=468) COVID-19: coronavirus disease 2019

Statement on lifestyle-related behavior	Before COVID-19 (frequency (%))	During COVID-19 (frequency (%))
Maintained a regular meal pattern		
Not routinely	87 (18.6)	94 (20.1)
1-2 times a week	55 (11.7)	62 (13.2)
3-4 times a week	95 (20.3)	100 (21.4)
5-6 times a week	63 (13.5)	61 (13)
Almost daily	168 (35.9)	151 (32.3)
Consumed fast food such as pizza, burgers, pasta, or noodles as snacks or meals		
Not routinely	287 (61.3)	328 (70.1)
1-2 times a week	147 (31.4)	106 (22.6)
3-4 times a week	23 (4.9)	26 (5.6)
5-6 times a week	5 (1.1)	3 (0.6)
Almost daily	6 (1.3)	5 (1.1)
Consumed fried food		
Not routinely	188 (40.2)	224 (47.9)
1-2 times a week	211 (45.1)	173 (37)
3-4 times a week	50 (10.7)	50 (10.7)
5-6 times a week	11 (2.3)	11 (2.3)
Almost daily	8 (1.7)	10 (2.1)
Consumed junk foods such as popcorn and chips		
Not routinely	269 (57.5)	306 (65.4)
1-2 times a week	151 (32.3)	111 (23.7)
3-4 times a week	30 (6.4)	35 (7.5)
5-6 times a week	9 (1.9)	8 (1.7)
Almost daily	9 (1.9)	8 (1.7)
Fruits and vegetable intake		
Not routinely	54 (11.5)	54 (11.5)
1-2 times a week	119 (25.4)	89 (19)
3-4 times a week	141 (30.1)	147 (31.4)
5-6 times a week	76 (16.3)	84 (18)
Almost daily	78 (16.7)	94 (20.1)
Had a balanced diet by including healthy ingredients (whole wheat, pulses, legumes, eggs, nuts, fruits, and vegetables) in meals		
Not routinely	58 (12.4)	60 (12.8)
1-2 times a week	103 (22)	95 (20.3)
3-4 times a week	132 (28.2)	117 (25)
5-6 times a week	78 (16.7)	91 (19.5)
Almost daily	97 (20.7)	105 (22.4)
Had 2-3 servings of milk or its products		
Not routinely	76 (16.2)	77 (16.5)
1-2 times a week	96 (20.5)	88 (18.8)
3-4 times a week	97 (20.7)	94 (20.1)
5-6 times a week	71 (15.2)	84 (17.9)
Almost daily	128 (27.4)	125 (26.7)
Pulses (dhal), eggs, or meat consumed in a day		
Not routinely	124 (26.5)	121 (25.9)
1-2 times a week	155 (33.1)	142 (30.3)
3-4 times a week	113 (24.1)	117 (25)
5-6 times a week	41 (8.8)	50 (10.7)
Almost daily	35 (7.5)	38 (8.1)
Teaspoons of sugar/honey/jaggery consumed in a day		
I do not add sugar	107 (22.9)	105 (22.4)
1-2 teaspoons per day	252 (53.8)	252 (53.8)
3-4 teaspoons per day	85 (18.2)	84 (18)
5-6 teaspoons per day	14 (3)	13 (2.8)
More than 6 teaspoons per day	10 (2.1)	14 (3)
Consumed sugar-sweetened beverages		
Not routinely	266 (56.9)	286 (61.1)
1-2 times a week	136 (29.1)	117 (25)
3-4 times a week	39 (8.3)	44 (9.4)
5-6 times a week	10 (2.1)	5 (1.1)
Almost daily	17 (3.6)	16 (3.4)
Consumed foods with high sugar such as sweet porridges, pastries, sweets, and chocolate		
Not routinely	257 (54.9)	279 (59.6)
1-2 times a week	144 (30.8)	129 (27.6)
3-4 times a week	47 (10)	40 (8.6)
5-6 times a week	13 (2.8)	10 (2.1)
Almost daily	7 (1.5)	10 (2.1)
Eat junk food due to boredom		
Not routinely	280 (59.8)	312 (66.6)
1-2 times a week	144 (30.8)	116 (24.8)
3-4 times a week	34 (7.2)	27 (5.8)
5-6 times a week	5 (1.1)	8 (1.7)
Almost daily	5 (1.1)	5 (1.1)
Participated in 30-minute exercises/sports		
Not routinely	224 (47.9)	226 (48.3)
1-2 times a week	112 (23.9)	106 (22.7)
3-4 times a week	63 (13.5)	65 (13.9)
5-6 times a week	33 (7)	32 (6.8)
Almost daily	36 (7.7)	39 (8.3)
Participated in household chores		
Not routinely	111 (23.7)	104 (22.2)
1-2 times a week	112 (23.9)	87 (18.6)
3-4 times a week	86 (18.4)	90 (19.2)
5-6 times a week	42 (9)	59 (12.6)
Almost daily	117 (25)	128 (27.4)
Participated in leisure activities such as grocery shopping, walking in the park, and gardening		
Not routinely	132 (28.2)	185 (39.5)
1-2 times a week	159 (34)	148 (31.6)
3-4 times a week	92 (19.7)	69 (14.8)
5-6 times a week	46 (9.8)	32 (6.8)
Almost daily	39 (8.3)	34 (7.3)
Daily sitting time at work		
Less than 2 hours	122 (26.1)	123 (26.3)
2-4 hours	111 (23.7)	104 (22.2)
4-6 hours	78 (16.7)	80 (17.1)
6-8 hours	119 (25.4)	110 (23.5)
More than 8 hours	38 (8.1)	51 (10.9)
Breaks from sitting at work		
Nil	48 (10.2)	70 (15)
1-2	189 (40.4)	163 (34.8)
3-4	116 (24.8)	109 (23.3)
5-6	65 (13.9)	64 (13.7)
More than 6	50 (10.7)	62 (13.2)
Screen time spent daily watching television and using mobile, laptop, or desktop		
0-1 hour	99 (21.2)	76 (16.3)
1-2 hours	173 (37)	103 (22)
2-4 hours	139 (29.7)	156 (33.3)
>5 hours	57 (12.2)	133 (28.4)
Hours slept daily		
<6 hours	79 (16.9)	71 (15.2)
6-8 hours	342 (73.1)	294 (62.8)
>8 hours	47 (10)	103 (22)
Quality of sleep (sound sleep without disturbance)		
Excellent	83 (17.7)	72 (15.4)
Very good	165 (35.3)	136 (29.1)
Good	198 (42.3)	214 (45.7)
Bad	20 (4.3)	38 (8.1)
Very bad	2 (0.4)	8 (1.7)
Stress or anxiety felt in a day		
Not at all	118 (25.2)	67 (14.3)
A little	265 (56.6)	197 (42.1)
Much	67 (14.3)	119 (25.4)
Very much	15 (3.2)	64 (13.7)
Extremely	3 (0.6)	21 (4.5)
Smoking habit		
No	437 (93.4)	441 (94.2)
Yes, 1-3 cigarettes per day	22 (4.7)	13 (2.8)
Yes, 4-6 cigarettes per day	8 (1.7)	13 (2.8)
Yes, 7-9 cigarettes per day	1 (0.2)	1 (0.2)
Drink alcohol		
No	384 (82.1)	394 (84.2)
Yes, on special occasions	63 (13.5)	51 (10.9)
Yes, on weekends	16 (3.4)	17 (3.6)
Yes, more than once a week	3 (0.6)	4 (0.9)
Yes, almost daily	2 (0.4)	2 (0.4)
Support in maintaining a healthy lifestyle		
Always (more than 90% of the time)	267 (57.1)	289 (61.8)
Most of the time (approximately 75% of the time)	102 (21.8)	83 (17.7)
Sometimes (approximately 50% of the time)	56 (12)	59 (12.6)
Occasionally (approximately 25% of the time)	25 (5.3)	17 (3.6)
Rarely (less than or equal to 10% of the time)	18 (3.8)	20 (4.3)

Comparison of mean score difference in lifestyle-related behavior during and before COVID-19 reveals that there was a significant reduction in the consumption of regular meals (0.12, SD: 0.96, p<0.05), fast food (0.10, SD: 0.71, p<0.05), junk foods (0.08, SD: 0.74, p<0.05), sweetened beverages (0.06, SD: 0.58, p<0.05), food with high sugar (0.06, SD: 0.60, p<0.05), and food to avoid boredom (0.07, SD: 0.72, p<0.05). Also, there was a significant reduction in participation in leisure activities (0.25, SD: 1.08, p<0.001) and friends and family support to maintain a healthy lifestyle (0.06, SD: 0.55, p<0.05).

There was a significant improvement found during COVID-19 in the intake of fruits and vegetables (0.15, SD: 0.88, p<0.001) and pulses (high-protein grains (daal)), egg, or meat (0.07, SD: 0.69, p<0.05). Participation in household chores (0.17, SD: 0.99, p<0.001), screen time in watching mobile or television (0.41, SD: 0.84, p<0.001), and stress or anxiety felt in a day (0.55, SD: 0.92, p<0.001) were significantly increased. Also, there was a significant improvement found in sleeping hours (0.13, SD: 0.63, p<0.001) and quality of sleep (0.17, SD: 0.77, p<0.001) (Table 3).

**Table 3:** Changes in lifestyle behavior before and during COVID-19 (N=468) #: mean difference score before and during COVID-19, *: significance at p<0.05, COVID-19: coronavirus disease 2019, SD: standard deviation

Statement on lifestyle-related behavior	Before COVID-19 pandemic (mean (SD))	During COVID-19 pandemic (mean (SD))	#Mean difference (SD)	t (p-value)
Eating behavior	26.84 (6.10)	26.64 (6.17)	0.20 (4.34)	
Maintained a regular meal pattern	3.36 (1.52)	3.24 (1.52)	0.12 (0.96)	2.73 (0.007)*
Consumed fast food	1.50 (0.75)	1.40 (0.72)	0.10 (0.71)	2.93 (0.004)*
Consumed fried food	1.80 (0.85)	1.74 (0.90)	0.06 (0.74)	1.87 (0.062)
Consumed junk foods	1.59 (0.85)	1.51 (0.84)	0.08 (0.74)	2.30 (0.022)*
Fruits and vegetable intake	3.01 (1.24)	3.16 (1.27)	0.15 (0.88)	3.70 (<0.001)*
Had a balanced diet with healthy ingredients	3.11 (1.31)	3.18 (1.33)	0.07 (0.86)	1.78 (0.076)
Had 2-3 servings of milk or its products	3.17 (1.44)	3.20 (1.44)	0.03 (0.83)	0.73 (0.469)
Had one or more servings of pulses, egg, or meat in a day	2.38 (1.18)	2.50 (1.21)	0.07 (0.69)	2.27 (0.024)*
Teaspoons of sugar/honey/jaggery consumed in a day	2.08 (0.85)	2.10 (0.88)	0.02 (0.54)	0.95 (0.344)
Consumed sugar-sweetened beverages	1.67 (0.98)	1.61 (0.95)	0.06 (0.58)	2.22 (0.027)*
Consumed foods with high sugar	1.65 (0.88)	1.60 (0.89)	0.06 (0.60)	2.01 (0.045)*
Eat junk food/fast food due to boredom/distress	1.53 (0.77)	1.46 (0.77)	0.07 (0.72)	2.13 (0.033)*
Physical activity	15 (3.93)	15.39 (4.12)	0.40 (3.12)	
Participated in 30 minutes of moderate-intensity aerobic exercises	2.03 (1.26)	2.04 (1.28)	0.02 (1.13)	0.29 (0.774)
Participated in household chores	2.88 (1.51)	3.04 (1.52)	0.17 (0.99)	3.66 (<0.001)*
Participated in leisure-related activities	2.36 (1.22)	2.11 (1.21)	0.25 (1.08)	5.08 (<0.001)*
Daily sitting time at work	2.66 (1.32)	2.71 (1.36)	0.05 (0.91)	1.12 (0.262)
Breaks from sitting	2.74 (1.15)	2.75 (1.25)	0.01 (0.81)	0.29 (0.776)
Screen time spent daily in watching television and using mobile	2.33 (0.94)	2.74 (1.04)	0.41 (0.84)	10.60 (<0.001)*
Sleep pattern	6.26 (1.38)	7.11 (1.72)	-0.85 (1.49)	
Hours slept daily	1.94 (0.52)	2.07 (0.61)	0.13 (0.63)	4.50 (<0.001)*
Quality of sleep	2.34 (0.83)	2.52 (0.91)	0.17 (0.77)	4.88 (<0.001)*
Stress or anxiety felt in a day	1.97 (0.76)	2.52 (1.04)	0.55 (0.92)	12.87 (<0.001)*
Other lifestyle behavior	4.10 (1.45)	4.02 (1.50)	0.08 (0.79)	
Smoking habit	1.09 (0.36)	1.09 (0.39)	0.00 (0.32)	0.14 (0.887)
Drink alcohol	1.24 (0.59)	1.22 (0.59)	0.02 (0.34)	0.94 (0.346)
Family and friends support maintaining a healthy lifestyle	1.77 (1.10)	1.71 (1.09)	0.06 (0.55)	2.44 (0.015)

Reasons for lifestyle changes during the COVID-19 pandemic

In comparison to pre-COVID-19 times, improved knowledge about nutrition (241 (51.5%)), lack of access to fresh fruits and vegetables (49 (10.5%)), and less eating out (36 (7.7%)) were the reasons for the changes in dietary patterns. The reasons for the changes in junk food/fast food consumption patterns in comparison to pre-COVID-19 times were fear of the spread of COVID-19 through food (183 (39.1%)), non-availability of cooks (31 (6.6%)), less eating out/socializing (79 (16.9%)), and preferring home-cooked foods (72 (15.4%)).

During COVID-19, the reasons for the change in the physical activity regimen were reported by participants as lack of motivation (109 (23.3%)), lack of access to sports facilities and gym (96 (20.5%)), social restrictions to parks and public places (111 (23.7%)), lack of social support (80 (17.1%)), and lack of time (51 (10.9%)). To increase physical activities, the participants included activities such as aerobics (179 (38.2%)), yoga (95 (20.3%)), and walks (87 (18.6%)); 50 (10.7%) participants reported not doing any activities.

The reasons for the change in sleeping patterns during COVID-19 were daytime sleeping (170 (36.3%)), stress and anxiety (102 (21.8%)), and long working hours (77 (16.5%)). The reasons for the change in the stress and anxiety levels of participants during COVID-19 were fear of COVID-19 infection (244 (52.1%)) and worrying about family and friends (112 (23.9%)) (Table 4).

**Table 4:** Reasons for lifestyle changes during the COVID-19 pandemic (N=468) COVID-19: coronavirus disease 2019

Reasons for lifestyle changes during the COVID-19 pandemic	Frequency (percentage)	
Reasons for changes in dietary patterns in comparison to pre-COVID-19 times		
Improved knowledge about nutrition		241 (51.5)
Lack of access to fresh fruits and vegetables		49 (10.5)
Reduced eating outside foods		36 (7.7)
Reasons for change in junk/fast food consumption in comparison to pre-COVID-19 times		
Fear of the spread of coronavirus through food		183 (39.1)
Non-availability of cooks		31 (6.6)
Less eating out/socializing		79 (16.9)
Preferring home-cooked food		72 (15.4)
Reasons for the change in physical activity regimen during COVID-19		
Lack of motivation		109 (23.3)
Lack of access to sports facilities and gym		96 (20.5)
Social restrictions to parks and public places		111 (23.7)
Lack of social support		80 (17.1)
Lack of time		51 (10.9)
Activities the participants included to increase physical activity during COVID-19		
Aerobics		179 (38.2)
Yoga		95 (20.3)
Walk		87 (18.6)
Not doing any activities		50 (10.7)
Reasons for the change in sleeping patterns during COVID-19		
Daytime sleeping		170 (36.3)
Stress and anxiety		102 (21.8)
Long working hours		77 (16.5)
Reasons for the change in the stress and anxiety level of participants during COVID-19		
Fear of COVID-19 infection		244 (52.1)
Worrying about family and friends		112 (23.9)

Association between lifestyle behaviors (before, during, total, and the difference between COVID-19 mean score) and demographic variables

Association Between Eating Behavior and the Demographic Characteristics of the Study Participants

There was a significant association found between eating behavior scores of participants and gender, marital status, nature of work, and current or past history of illness. The eating behavior scores of the participants before COVID-19, during COVID-19, and in total were higher in females than in males at 2.469 (0.014), 2.815 (0.005), and 2.833 (0.005), respectively. Healthy eating behavior was found in participants who were divorced/unmarried (2.142 (0.033)) and without a history of past/present illness (2.076 (0.044)) before COVID-19. There was a significant association between eating behavior lifestyle score and nature of work during COVID-19 (3.017 (0.011)) and in total (2.642 (0.023)). Field workers tended to have healthier eating habits than long-standing (3.277 (0.024)) and sedentary/computer (3.821 (0.016)) workers during the pandemic. The total mean eating lifestyle behavior score of field workers was higher than long-standing (5.849 (0.035)) and sedentary/computer (6.420 (0.043)) workers.

Association Between Physical Activity and the Demographic Characteristics of the Study Participants

Before COVID-19, a significant association was found between physical activity score and type of residence (6.900 (0.001)) and hours of work (10.724 (<0.001)). During COVID-19, a significant association was found between physical activity and gender (2.410 (0.016)), residence (7.327 (0.001)), nature of work (2.993 (0.011)), and hours of work (8.534 (<0.001)) of participants. The total physical activity score of participants was significantly associated with their residence (8.409 (<0.001)) and hours of work (10.994 (<0.001)). The difference in physical activity score of participants was significantly associated with their type of family (2.782 (0.006)) and nature of work (2.890 (0.014)). During COVID-19, higher physical activity was found in females (2.410 (0.016)) and field workers as compared to sedentary/computer workers (2.822 (0.005)) and other workers (2.578 (0.022)). The physical activity life score was higher in field workers (1.951 (0.022)) and office workers (1.450 (0.040)) than in others. Before COVID-19, rural (1.474 (0.002)) and semi-urban (1.146 (0.036)) residents had higher physical activity than urban residents, whereas during COVID-19, the score was 1.620 (0.001) and 1.182 (0.040), respectively. In relation to total physical activity score, urban residents had lesser physical activity than rural (3.094 (0.001)) and semi-urban (2.328 (0.021)) residents. Before COVID-19, the participants’ working hours were either six hours (2.508 (<0.001)) or 6-8 hours (1.621 (<0.001)) rather than >8 hours of work per day. During COVID-19, the participants worked six hours (1.794 (<0.001)) or 6-8 hours (1.804 (<0.001)) rather than >8 hours of work per day. The total mean physical activity score shows that participants who worked more than eight hours were doing fewer physical activities than those who worked for six hours (4.312 (<0.001)) and 6-8 hours (3.414 (<0.001)) per day.

Association Between Sleeping Behavior and the Demographic Characteristics of the Study Participants

A significant association was found between sleeping patterns and the gender of participants. Before COVID-19, females had better sleeping patterns than males (3.109 (0.002)), and during COVID-19, male participants had significant improvement in sleeping patterns (2.443 (0.015)) than females.

Association Between Other Lifestyle Behaviors and the Demographic Characteristics of the Study Participants

Before COVID-19, other lifestyle behaviors were higher in those 31-60 years of age (2.350 (0.020)), males (2.180 (0.030)), those qualified as graduate (2.076 (0.040)), and those with a current or past history of chronic disease (2.624 (0.012)). During COVID-19, other lifestyle behaviors were higher in participants aged 31-60 years (1.977 (0.049)) and those with a current or past history of chronic disease (2.697 (0.010)). The association between total scores related to other lifestyle behaviors shows that there was a significant increase in other lifestyle behaviors among those 31-60 years (2.221 (0.027)), males (2.158 (0.032)), and those who have chronic past/present illness (2.703 (0.010)). A significant association was found between hours of work and other lifestyle behaviors before COVID-19 (3.082 (0.047)) and in total (3.123 (0.045)). Before COVID-19 (0.393 (0.036)) and in total (0.777 (0.034)), other lifestyle behaviors were higher in those working for 6-8 hours than those working for eight hours per day. There was a significant association found between other lifestyle behaviors and the residence of the participants before COVID-19 (4.896 (0.008)), in total (3.351 (0.036)), and in the difference in score between before and during COVID-19 (3.328 (0.037)). Before COVID-19, participants who lived in rural areas had higher other lifestyle behaviors than those who lived in semi-urban areas (0.588 (0.009)) and urban areas (0.407 (0.033)). The total other lifestyle behavior score reflects that participants in rural areas had higher other lifestyle behaviors than participants in semi-urban areas (0.995 (0.030)). The difference in lifestyle behavior scores before and during COVID-19 shows that participants in rural areas had higher other lifestyle behaviors than participants in urban areas (0.228 (0.028)) (Table 5).

**Table 5:** Association between lifestyle behaviors (before, during, total, and the difference between COVID-19 mean score) and demographic variables *: significance at p<0.05, t: independent t-test value, F: analysis of variance value, COVID-19: coronavirus disease 2019, SD: standard deviation

Lifestyle behavior/demographic variable	Age	Gender	Educational status	Marital status	Type of family	Type of residence	Nature of work	Hours of work	Current/past chronic illness
	t (p-value)	t (p-value)	t (p-value)	t (p-value)	t (p-value)	F (p-value)	F (p-value)	F (p-value)	t (p-value)
Eating behavior									
Before COVID-19	1.065 (0.288)	-2.469 (0.014)*	-.236 (0.813)	-2.142 (0.033)*	1.348 (0.178)	.016 (0.984)	2.036 (0.072)	.349 (0.706)	-2.076 (0.044)*
During COVID-19	0.699 (0.485)	-2.815 (0.005)*	-0.614 (0.539)	-1.530 (0.127)	0.934 (0.351)	1.036 (0.356)	3.017 (0.011)*	0.496 (0.609)	-1.265 (0.206)
Total mean (SD)	0.942 (0.347)	-2.833 (0.005)*	-0.456 (0.649)	-1.932 (0.054)	1.218 (0.224)	0.368 (0.693)	2.642 (0.023)*	0.471 (0.625)	-1.895 (0.059)
Mean difference (SD)	0.501 (0.617)	0.578 (0.563)	0.542 (0.588)	0.749 (0.454)	0.565 (0.573)	1.683 (0.187)	1.749 (0.122)	0.089 (0.915)	-1.404 (0.161)
Physical activity									
Before COVID-19	-1.035 (0.301)	-1.003 (0.316)	-1.787 (0.075)	1.185 (0.237)	-1.945 (0.052)	6.900 (0.001)*	1.061 (0.381)	10.724 (<0.001)	-1.520 (0.129)
During COVID-19	-0.682 (0.496)	-2.410 (0.016)*	-1.478 (0.140)	0.281 (0.779)	0.209 (0.835)	7.327 (0.001)*	2.993 (0.011)*	8.534 (<0.001)	-0.808 (0.419)
Total mean (SD)	-0.926 (0.355)	-1.889 (0.060)	-1.767 (0.078)	0.782 (0.434)	-0.910 (0.363)	8.409 (<0.001)	1.919 (0.090)	10.994 (<0.001)	-1.253 (0.211)
Mean difference (SD)	-0.402 (0.688)	1.963 (0.050)	-0.295 (0.768)	1.121 (0.263)	-2.782 (0.006)*	0.086 (0.918)	2.890 (0.014)*	2.077 (0.126)	-0.844 (0.399)
Sleep pattern									
Before COVID-19	-0.016 (0.987)	-3.109 (0.002)*	-1.324 (0.186)	-0.983 (0.326)	0.884 (0.377)	1.841 (0.160)	1.131 (0.343)	0.626 (0.535)	1.429 (0.154)
During COVID-19	-1.096 (0.274)	-0.311 (0.756)	-1.029 (0.304)	0.539 (0.590)	0.962 (0.336)	1.873 (0.155)	1.278 (0.272)	1.407 (0.246)	0.310 (0.756)
Total mean (SD)	-0.696 (0.487)	-1.760 (0.079)	-1.313 (0.190)	-0.156 (0.876)	1.050 (0.294)	2.364 (0.095)	1.385 (0.229)	1.304 (0.272)	0.914 (0.361)
Mean difference (SD)	1.250 (0.212)	-2.443 (0.015)*	-0.036 (0.971)	-1.533 (0.126)	-0.292 (0.770)	0.177 (0.838)	0.671 (0.645)	0.419 (0.658)	0.962 (0.337)
Other lifestyle behaviors									
Before COVID-19	-2.350 (0.020)*	2.180 (0.030)*	-2.076 (0.040)*	1.828 (0.068)	0.351 (0.726)	4.896 (0.008)*	1.169 (0.323)	3.082 (0.047)*	2.624 (0.012)*
During COVID-19	-1.977 (0.049)*	1.967 (0.050)	-1.537 (0.125)	1.263 (0.207)	-0.164 (0.870)	1.928 (0.147)	0.473 (0.797)	2.730 (0.066)	2.697 (0.010)*
Total mean (SD)	-2.221 (0.027)*	2.158 (0.032)*	-1.846 (0.066)	1.599 (0.111)	0.092 (0.927)	3.351 (0.036)*	0.732 (0.600)	3.123 (0.045)*	2.703 (0.010)*
Mean difference (SD)	-0.630 (0.529)	0.259 (0.796)	-0.788 (0.431)	0.940 (0.348)	0.952 (0.341)	3.328 (0.037)*	1.785 (0.114)	0.052 (0.949)	-0.382 (0.702)

## Discussion

Lifestyle behaviors are everyday activities that result from an individual’s values, knowledge, and norms shaped by broader cultural and socioeconomic contexts. These behaviors affect an individual’s overall health and are influenced by several social characteristics. The present study revealed the impact of the COVID-19 outbreak on lifestyle-related behavior among the general population. Of all the participants, 57.7% were females, and 42.3% were males. The majority of the study participants (44.4%) had a stable weight during COVID-19. This finding is in agreement with the study conducted by Scarmozzino and Visioli [12], Zachary et al. [13], Fernandez-Rio et al. [14], Reyes-Olavarría et al. [15], and Al-Musharaf et al. [16], where most of the participants had a stable weight during the COVID-19 pandemic. However, a study by Al-Domi et al. [17] found an increase in body weight among most of its participants.

The present study revealed a decrease in the consumption of fast food, junk food, and fried food. The reason for the same was less eating out/socialization, fear of the spread of COVID-19, and non-availability of cooks. Interestingly, the present study showed an increase in the intake of fruits and vegetables (3.70 (<0.001)). Similar findings were seen in a study conducted by Scarmozzino and Visioli [12], in which 21.2% of respondents increased their fruit and vegetable intake, and the purchase of ready-made snacks was reduced by 50%. In a study conducted by Chopra et al. [18], an improvement in healthy meal consumption patterns and a restriction of unhealthy food items were observed. Contrary to this, a study done by Werneck et al. [19] reported less consumption of fruits or vegetables and more ultra-processed foods.

The present study shows decreased participation in leisure-related activities (5.08 (<0.001)), and an increase in screen time spent daily, e.g., watching television and mobile phones (10.60 (<0.001)). Similar findings were seen in a study done by Chopra et al. [18], Werneck et al. [19], and Cheikh Ismail et al. [20], where the majority of participants reported having more screen time and less physical activity.

The current study also investigated the impact of the COVID-19 pandemic on sleep patterns, which revealed an increase in sleeping hours, as well as the quality of sleep, which is similar to the findings of Al-Musharaf et al. [16]. The study done by Marelli et al. [21] showed an increase in sleep time, but the quality of sleep was found to be poor.

The stress or anxiety the participants felt in a day was also found to be significantly increased (12.87 (<0.001)) in the present study. The reason for the same was reported by study subjects as fear of COVID-19 infection and concern about family and friends. A systematic review and meta-analysis by Salari et al. [22] also revealed similar findings.

Strengths of the study

The area under study is less explored by other researchers, and a standardized tool is used in the study, which shows its credibility.

Limitations of the study

Data were collected using Google Forms, so a few participants experienced technical difficulties when attempting to answer the questionnaire. Also, due to the convenience sampling technique, the findings of this study can not be generalized to the entire Indian population. The lifestyle behavior of mankind is a complex phenomenon. Researchers have tried to explore information on selected domains of lifestyle behavior, viz., eating habits, sleep patterns, and consumption of alcohol. Further research can be done on various other domains of interest that could have been affected or changed by the COVID-19 pandemic (e.g., cultural influence). Additionally, more intensive information through a phenomenological research approach can be explored. This data will help identify and mitigate any negative lifestyle behavior. Public health promotion platforms can also be installed to provide one point of solution for any guidance or support to enhance a positive and healthy lifestyle behavior during the present or possible future pandemic.

## Conclusions

The recent COVID-19 pandemic has influenced individual lifestyle behavior and preferences globally. Much can be attributed to lockdown, other imposed restrictions, and the availability of resources. The present study findings indicate a positive lifestyle behavior in certain aspects such as healthy food intake, participation in household chores, marginal reduction in alcohol consumption and smoking, and improvement in sleep patterns. This study also showed an increase in screen time spent and no significant improvement in time spent on physical exercises. The anxiety experienced by the participants was also reported to be increased. A detailed study on further exploration of ways to improve and sustain healthy behavior can help promote health and a positive lifestyle.

## References

[1] Ferrante G, Camussi E, Piccinelli C (2020). Did social isolation during the SARS-CoV-2 epidemic have an impact on the lifestyles of citizens?. Epidemiol Prev.

[2] Husain W, Ashkanani F (2020). Does COVID-19 change dietary habits and lifestyle behaviours in Kuwait: a community-based cross-sectional study. Environ Health Prev Med.

[3] Ammar A, Brach M, Trabelsi K (2020). Effects of COVID-19 home confinement on eating behaviour and physical activity: results of the ECLB-COVID19 International Online Survey. Nutrients.

[4] Rawat D, Dixit V, Gulati S, Gulati S, Gulati A (2021). Impact of COVID-19 outbreak on lifestyle behaviour: a review of studies published in India. Diabetes Metab Syndr.

[5] Fanelli RM (2021). Changes in the food-related behaviour of Italian consumers during the COVID-19 pandemic. Foods.

[6] Zhu S, Zhuang Y, Ip P (2021). Impacts on children and adolescents’ lifestyle, social support and their association with negative impacts of the COVID-19 pandemic. Int J Environ Res Public Health.

[7] Monye I, Adelowo AB (2020). Strengthening immunity through healthy lifestyle practices: recommendations for lifestyle interventions in the management of COVID‐19. Lifestyle Med.

[8] Di Renzo L, Gualtieri P, Pivari F (2020). Eating habits and lifestyle changes during COVID-19 lockdown: an Italian survey. J Transl Med.

[9] (2020). Boosting your immune system against coronavirus: how to minimize the risk of infection?. https://www.narayanahealth.org/blog/boost-immune-system-against-coronavirus-covid-19-infection/.

[10] (2021). How to boost your immune system. Harvard Health Publishing.

[11] Chopra S, Ranjan P, Malhotra A (2021). Development and validation of a questionnaire to evaluate the impact of COVID-19 on lifestyle-related behaviours: eating habits, activity and sleep behaviour. Public Health Nutr.

[12] Scarmozzino F, Visioli F (2020). COVID-19 and the subsequent lockdown modified dietary habits of almost half the population in an Italian sample. Foods.

[13] Zachary Z, Brianna F, Brianna L, Garrett P, Jade W, Alyssa D, Mikayla K (2020). Self-quarantine and weight gain related risk factors during the COVID-19 pandemic. Obes Res Clin Pract.

[14] Fernandez-Rio J, Cecchini JA, Mendez-Gimenez A, Carriedo A (2020). Weight changes during the COVID-19 home confinement. Effects on psychosocial variables. Obes Res Clin Pract.

[15] Reyes-Olavarría D, Latorre-Román PÁ, Guzmán-Guzmán IP, Jerez-Mayorga D, Caamaño-Navarrete F, Delgado-Floody P (2020). Positive and negative changes in food habits, physical activity patterns, and weight status during COVID-19 confinement: associated factors in the Chilean population. Int J Environ Res Public Health.

[16] Al-Musharaf S, Aljuraiban G, Bogis R, Alnafisah R, Aldhwayan M, Tahrani A (2021). Lifestyle changes associated with COVID-19 quarantine among young Saudi women: a prospective study. PLoS One.

[17] Al-Domi H, Al-Dalaeen A, Al-Rosan S, Batarseh N, Nawaiseh H (2021). Healthy nutritional behavior during COVID-19 lockdown: a cross-sectional study. Clin Nutr ESPEN.

[18] Chopra S, Ranjan P, Singh V (2020). Impact of COVID-19 on lifestyle-related behaviours- a cross-sectional audit of responses from nine hundred and ninety-five participants from India. Diabetes Metab Syndr.

[19] Werneck AO, Silva DR, Malta DC (2021). Associations of sedentary behaviours and incidence of unhealthy diet during the COVID-19 quarantine in Brazil. Public Health Nutr.

[20] Cheikh Ismail L, Osaili TM, Mohamad MN (2020). Eating habits and lifestyle during COVID-19 lockdown in the United Arab Emirates: a cross-sectional study. Nutrients.

[21] Marelli S, Castelnuovo A, Somma A (2021). Impact of COVID-19 lockdown on sleep quality in university students and administration staff. J Neurol.

[22] Salari N, Hosseinian-Far A, Jalali R (2020). Prevalence of stress, anxiety, depression among the general population during the COVID-19 pandemic: a systematic review and meta-analysis. Global Health.

